# A Treatise on the Diseases of the Chest

**Published:** 1852-08

**Authors:** 


*A Treatise on the Diseases of the Chest; being a Course of Lectures delivered at the New York Hospital.* By JOHN A. SWETT, M. D., Physician to the New York Hospital; Member of the New York Pathological Society. New York: D. Appleton and Company, 200 Broadway. 1852. 8vo. pp. 585.

Some ten years since, a series of lectures on the diseases of the chest, by Dr. Swett, were published in the *New York Lancet*, a weekly medical journal, issued at that time at New York, which has since been discontinued. They were received with marked favor by the medical profession, and served at once to establish the reputation of Dr. Swett as an able practitioner and teacher in the department of physical diagnosis. Upon these lectures the treatise, the title-page of which is prefixed, is based, the author yielding to the repeated solicitations of his medical friends to publish them in a separate volume. In the present republication not only has the subject been revised, but the experience of ten years of hospital and private practice has been added. The author states that during most of his professional life he has kept a register of the most important cases of chest disease that have fallen under his notice, and the materials therein contained have been freely used in the preparation of this work. "The statements," to quote the language of the preface, "are founded upon registered and carefully observed facts, rather than upon vague recollections." This recommendation of the work will be duly estimated by those who appreciate the wide difference between the loose hap-hazard assertions of those who draw their conclusions from recollected experience, and the results deduced by a rigorous analysis of recorded data. The former originate and give currency to a host of false facts to be slowly and with difficulty corrected by the labors of the careful observer and analyst. In an appendix to the treatise, Dr. Swett has given a translation from Lebert's work on pathological physiology, containing the discoveries of that distinguished microscopist in the structure of tubercle and of cancer, with well executed illustrations.

We have held this work in reserve for some time, designing to give it extended analytical reviewal; and it is with considerable reluctance that we feel obliged to forego the execution of that intent. As an American, and we may add a New York book, it claims thus much at our hands, but we find that the design must either be abandoned, or indefinitely postponed, and it is an injustice both to the author and our readers that the work should continue longer to lie on our table unnoticed.

The scope and character of the treatise will be understood from what has been already stated. The name of the author is too well known to require aught from us on that point. We have only to add, that after a tolerably attentive examination, we would warmly commend it to the perusal and study of our professional brethren. In saying this, it is hardly necessary to remark that we do by no means hold ourselves committed to all the views contained in the work. This is a disclaimer which we often take occasion to repeat, in order to avoid misconception. In point of fact, while perusing Dr. Swett's treatise, having in contemplation a critical review, we had marked several portions respecting which we should venture to differ from the able author. One of these we cannot forbear referring to in this brief notice. Speaking of the physical signs of early tuberculosis, Dr. S. says with respect to percussion, "posteriorly no advantage can be derived from percussion; the muscles are too thick about the shoulders to admit of any decided advantage even in cases in which the pulmonary condensation is much greater than in incipient phthisis." (Page 258.) We are greatly surprised at this statement by so skillful an auscultator as we know Dr. S. to be. Our own experience leads us to a different opinion. We have not infrequently met with cases in which a marked disparity in the sonoriety of the percussion sound was apparent over the scapular region, above and below the spinous ridge, when the resonance was nearly or quite equal in the infra clavicular regions, and under circumstances in which the fact was an important physical sign in determining the presence of tubercle. If attention be paid to the pitch, as well as amount of resonance, we are almost prepared to say that the scapular region is but little less important than the infra clavicular in seeking by percussion for the physical evidences of tubercle. This is a matter of sense and tact in which, it is evident, the appeal must be, not to argument, but demonstration.

In treating of diseases of the heart we are gratified to find that the author simplifies the subject by including the different varieties of hypertrophy and dilatation under a single class, to which he applies the simple term, enlargement. This course renders the subject much less complicated for the student, a point of considerable importance with respect, not only to these, but all other forms of disease. In giving public instruction we have been led to pursue a similar plan. Having thus alluded to this part of the work devoted to diseases of the heart, we cannot avoid expressing surprise that the author omits to refer to the fact that pericarditis is occasionally masked by cerebral symptoms which, without knowledge of this fact, will be almost sure to lead the practitioner to regard the brain as the seat of the disease. Cases illustrating this important point have been contributed by Watson, Burrows, and others, and a striking instance was reported by the Editor of this Journal in the April No., 1850. An instance somewhat similar has more recently fallen under our observation in which the diagnosis of pericarditis was predicated on sudden death taken in connection with the character of the cerebral symptoms, the previous history being unknown, and the patient dying before a careful examination of the case was made.

Assuming the validity of these, and other criticisms which we should presume to offer were we to enter deliberately on the task of a review, the merits of the work (which are generally left for the reader to discover without the aid of the critic) are such that, in mercantile phraseology, the balance in its favor should satisfy all reasonable claims of authorship.

The style of the work is lucid, but plain to a degree which sometimes seems to denote, not only neglect, but contempt for elegance of diction. We would not quarrel with the author on this score, and yet, we think attention to composition is not without its advantage even in a scientific production.

We should have preferred, also, to have had the work divided into sections and chapters, rather than into lectures, with a corresponding adaptation of style to the former arrangement. We know not how it strikes others, but whenever we sit down to read a volume of lectures, we cannot divest ourselves of the idea that we are stationed behind the door, or in an adjoining apartment, and listening, somewhat clandestinely, to words addressed, not to ourselves, but to others.

In taking leave of Dr. S. we would express the hope that a second edition of this treatise will be speedily demanded.

